# CD20-positive subcutaneous panniculitis-like T-cell lymphoma presenting as polycranial neuropathy: A CARE-compliant case report and literature review

**DOI:** 10.1097/MD.0000000000030233

**Published:** 2022-09-02

**Authors:** Jing Xu, Jia Li, Ya-juan Sun, Wei Quan, Li Liu, Qing-hui Zhang, Yi-dan Qin, Xiao-chen Pei, Hang Su, Jia-Jun Chen

**Affiliations:** a Department of Neurology, China–Japan Union Hospital of Jilin University, Jilin, China

**Keywords:** CD20, central nervous system, cranial nerve diseases, subcutaneous panniculitis-like T-cell lymphoma

## Abstract

Subcutaneous panniculitis-like T-cell lymphoma(SPTCL) is a very rare cytotoxic T-cell skin lymphoma involving subcutaneous tissue, and mainly affects young females. T-cell phenotype is characterized by CD3+, CD8+, and CD4−. SPTCT with polycranial neuropathy has rarely been described. SPTCL is believed to show an indolent clinical course unless patients develop haemophagocytic syndrome or sudden respiratory failure. Its treatment has not been established yet.

**Case presentation:**

We report a case of intractable SPTCT in a 66-year-old woman with multiple cranial nerve palsies and diabetes. She showed involvement of the bilateral facial nerve, left trigeminal nerve, left auditory nerve, and right oculomotor nerve. The single inconspicuous skin lesion in the trunk presented with an erythematous nodule with a diameter of <5 cm and a slightly pink infiltrated plaque. Electromyography revealed bilateral damage to the facial nerve. Differential immunohistochemical characteristics were observed. Immunohistochemistry demonstrated diffuse CD20 positivity. Cerebral spinal fluid analysis revealed elevated protein levels of 0.92 (0.15–0.45) g/L. Her condition regressed severely over time. She was treated with chemotherapy but died 10 months later, the probable cause of death was lung involvement.

**Conclusion:**

The patient’s involvement with the central nervous system may be associated with positivity for CD20. Molecular biomarkers may act as therapeutic targets for SPTCL.

## 1. Introduction

In 2005, based on the differential expression of the T-cell phenotype (αβ or γδ) and clinical features, the World Health Organization-European Organization for Research and Treatment of Cancer Classification for cutaneous lymphomas changed the term subcutaneous panniculitis-like T-cell lymphoma (SPTCL) to α/β T-cell phenotype SPTCL.^[1]^ SPTCL is a very rare cytotoxic T-cell lymphoma of the skin, clinically mimics panniculitis, and mainly affects young females.^[2–4]^ It mostly involves the lower extremities or trunk and manifests as multiple or single erythema nodules with an unidentifiable feeling, with early misdiagnosis rate being very high.^[5–7]^ As the disease progresses, the lesions gradually expand, measure from 0.5 cm to more than 10 cm,^[5–7]^ and even involve intra-abdominal fat,^[5]^ lung,^[8]^ bone marrow,^[8]^ cerebrum.^[9,10]^ In addition, there are some differences in the immunohistochemical staining of the tumors. The involvement of the central nervous system (CNS) in the SPTCL is rare.^[9,10]^ We present a case of SPTCL beginning as polycranial neuropathy that presented in a single anatomic site and a single skin lesion that involved the CNS and discuss clinical characteristics, immunohistochemical features, and literature review on SPTCL.

## 2. Case presentation

**Figure 1.**
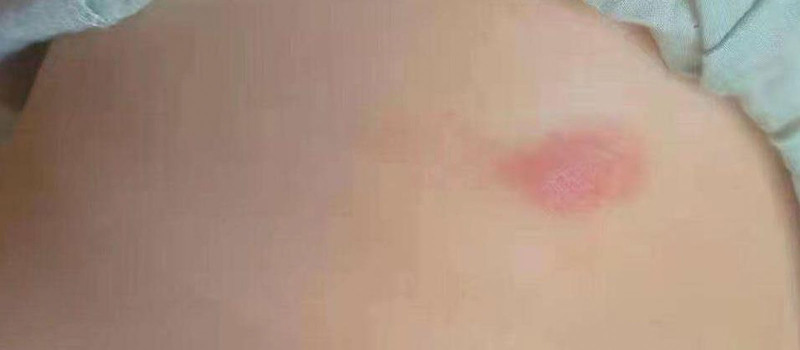
Singular, erythematous, indurated nodular lesions with a diameter of <5 cm localized on the trunk with no skin surface desquamation or ulceration.

**Figure 2.**
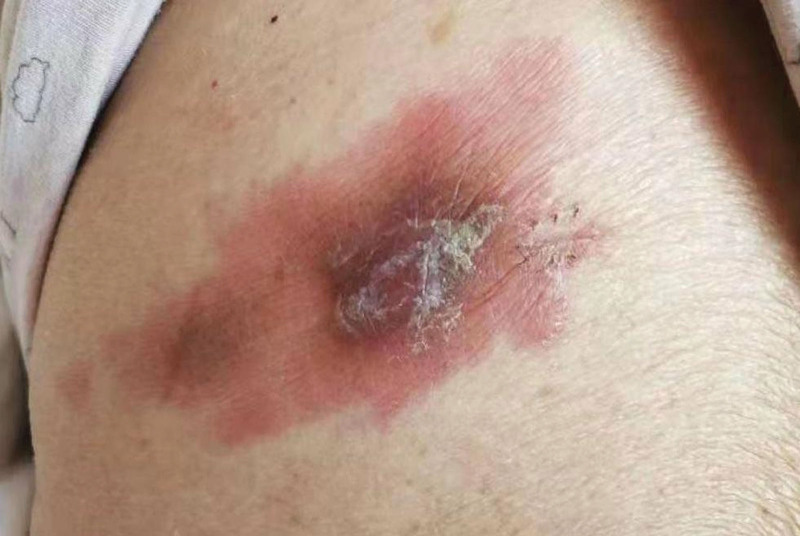
The erythema expands into patches, with obvious dark red nodules and white dandruff visible in the center. The boundaries are relatively clear.

This study was retrospective, the patient’s daughter provided written informed consent. In May 2020, a 66-year-old ethnic Han Chinese woman initially presented with left-side oral and facial numbness and hypesthesia. In September 2020, she presented with left facial nerve palsy and decreased hearing in her left ear. She also had a singular erythematous, indurated nodular lesion with a diameter of <5 cm localized on the trunk. There is no skin surface desquamation or ulceration (Fig. 1). The skin lesion was neglected. She was found to have had severe diabetes as evidenced by a glycated hemoglobin of 20.54% (4%–6%). Her symptoms did not improve by administering vitamin B1, vitamin B12, diabetic treatment, and other nutritional nerve treatments. Her condition regressed severely over time, and in December 2020, she presented with right facial nerve palsy where the right eye could not open, severely decreased hearing in the left ear, whole body fatigue, weight loss, and erythematous, indurated nodular lesion expansion (Fig. 2). She underwent a systematic examination on December 5, 2020. Physical examination revealed bilateral peripheral facial palsy, where the palsy on the right side was severe and the right eye could not be opened independently. On the left side, there was a mild impairment in the ability to sense a pinprick and light touch and mild hearing loss in the left ear. In addition, there were expanding patch-shaped erythematous nodules in the trunk. She had no medical, family, or psycho-social history or relevant genetic information.

**Figure 3.**
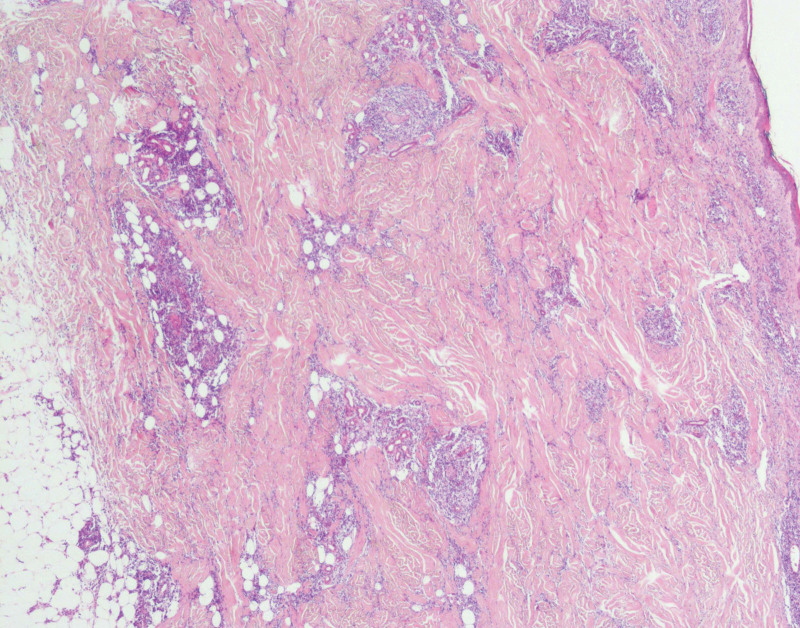
Biopsy of the skin lesion on the trunk revealed atypical lymphoid infiltration of subcutaneous fat with destruction of adipocytes, and extension to the dermis, blood vessels, and collagen fibrils (H&E staining, ×20).

**Figure 4.**
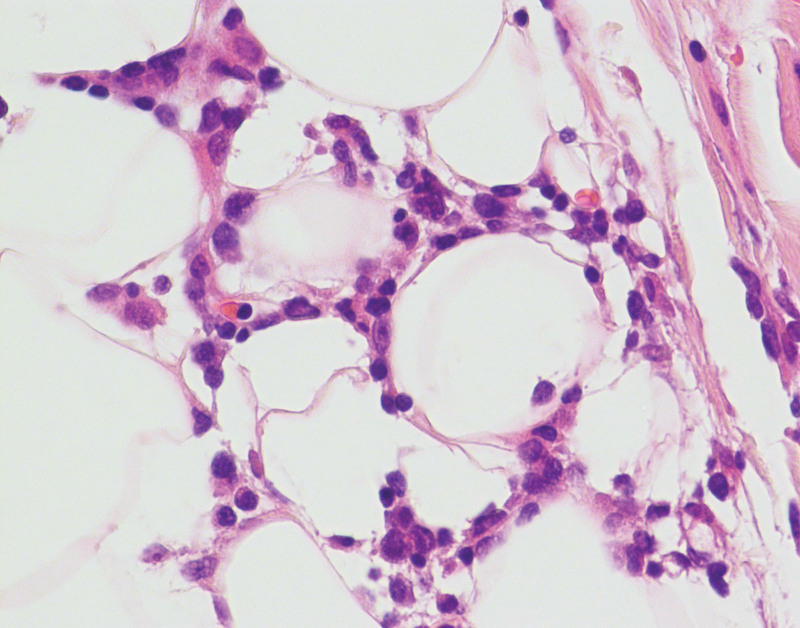
Atypical lymphocytes and associated histiocytes that are rimming the adipocytes, in a lace-like manner resembling panniculitis. The neoplastic infiltrate was composed of pleomorphic T cells with irregular and hyperchromatic nuclei (H&E staining, ×400).

**Figure 5.**
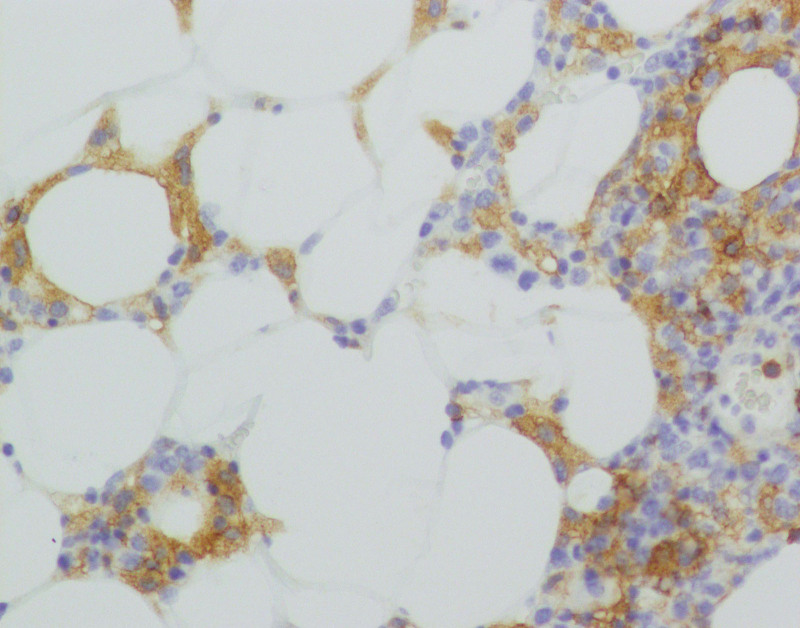
Immunohistological staining showing positivity for CD4 × 200.

**Figure 6.**
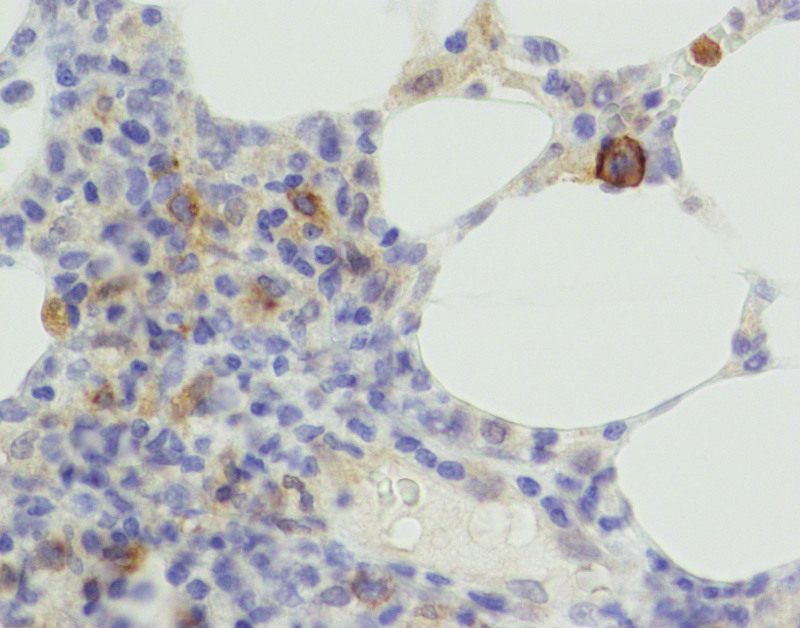
Immunohistological staining showing positivity for CD30 × 200.

**Figure 7.**
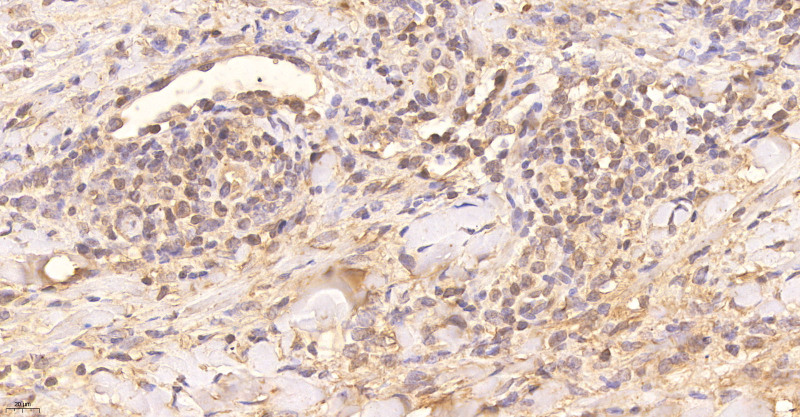
Immunohistological staining showing positivity for TCR-βF1 × 400.

**Figure 8.**
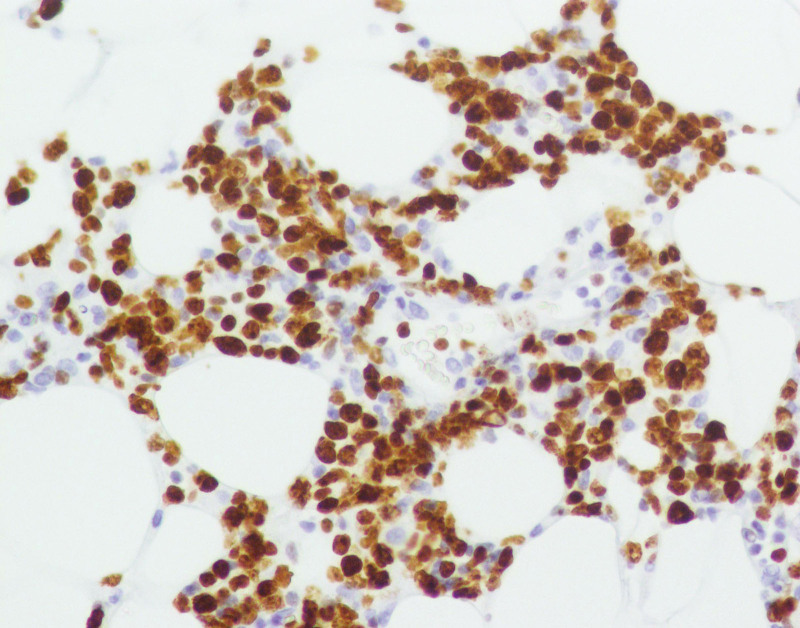
Immunohistological staining showing positivity for Ki67 × 200.

**Figure 9.**
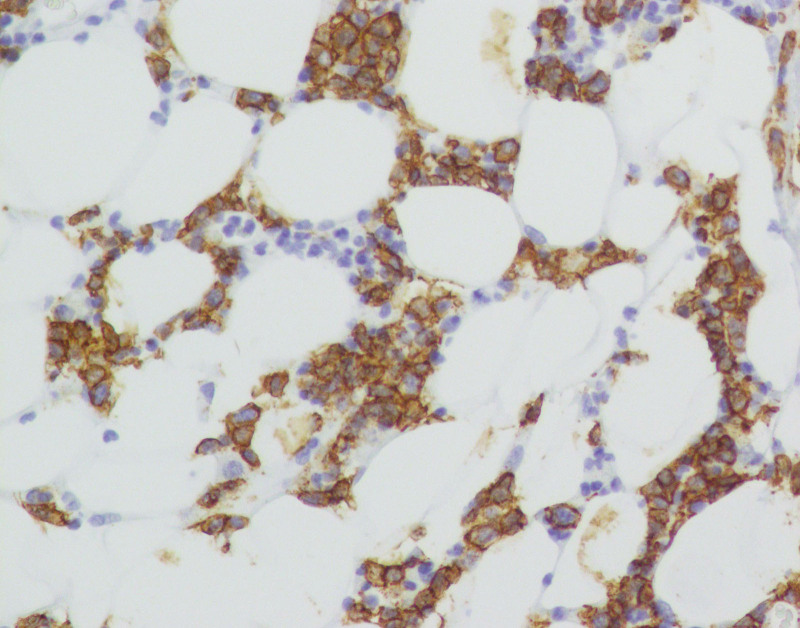
Immunohistological staining showing positivity for CD20 × 200.

**Figure 10.**
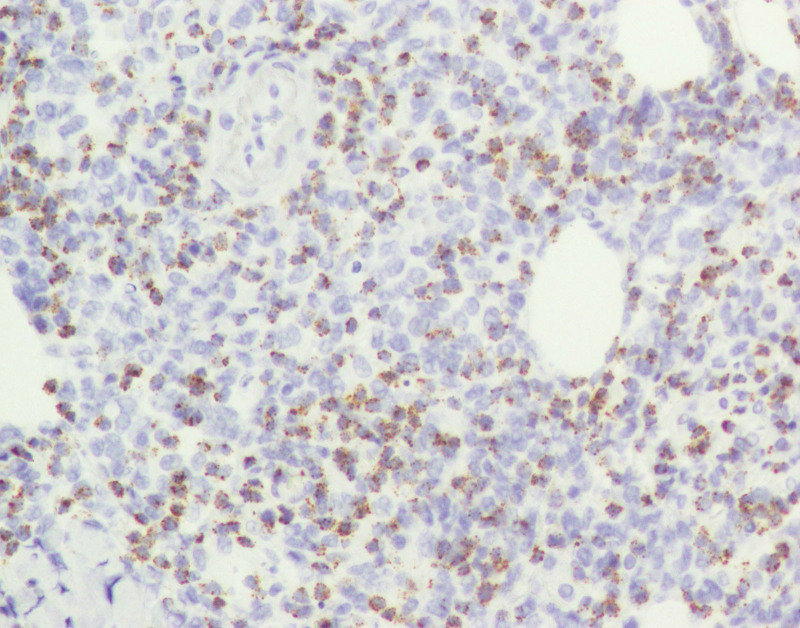
Immunohistological staining showing positivity for TiA1 × 200.

The following laboratory test results were either normal or negative: complete blood count, erythrocyte sedimentation rate, blood chemistry panel, tumoral markers, anti-human immunodeficiency virus antibodies, anti-hepatitis C virus antibodies, treponema pallidum antibody, antibodies against Epstein-Barr virus, coagulation function, antinuclear antibodies, antineutrophil cytoplasmic antibodies, Russell’s viper venom time ratio of lupus anticoagulation, anticardiolipin antibody, anti-β2-glycoprotein-1 antibodies, anti-myeloperoxidase antibodies, anti-proteinase 3 antibodies and anti-glomerular basement membrane antibodies, ρ anti-neutrophil cytoplasmic antibodies, c anti-neutrophil cytoplasmic antibodies, serum complement and immunoglobulins, β2 microglobulin, and Th1/Th2 cytokines. Findings of ultrasonography, chest/abdominal and pelvic computed tomography, head diffusion-weighted imaging, and magnetic resonance imaging were negative or normal. No findings were suggesting a hemophagocytic syndrome. Electromyography indicated bilateral facial nerve damage. The histopathologic examination of the lesion in the trunk revealed atypical lymphoid infiltration of the dermis and subcutaneous tissue, rimming of individual adipocytes by atypical lymphocytes and histiocytes, fat necrosis, and karyorrhexis. Extension of the atypical infiltrate into the dermis and infiltrating collagen fibrils were observed. Angioinvasion was indicated by banded intensive lymphocyte infiltrate around the blood vessels. The neoplastic infiltrate was composed of dense pleomorphic T cells with clear cytoplasm and hyperchromatic nuclei, which was admixed with small reactive lymphocytes and numerous histiocytes, and a single nuclear type (Figs. 3,4). The T-cells were positive for CD2, CD3, CD4, CD5, CD8, CD20, Ki67 (85%), cytotoxic proteins TiA1, CD30, CD31, T-cell receptor (TCR)-βF1 and negative for CD123, CD7, CD56, and Epstein–Barr virus-encoded small RNA. Figs. 5–10 shows immunohistological staining positivity for CD4, CD30, TCR-βF1, Ki67, CD20, and TiA1. T-cell gene rearrangement (paraffin-embedded formalin-fixed skin tissue) of TCR-β, TCR-γ, and TCR-δ by polymerase chain reaction failed to demonstrate clonality. Bone marrow aspiration smears showed that the proportion of lymphoid cells increased. Immunohistochemical staining of bone marrow biopsy showed no abnormal immunophenotype, no hemophagocytosis, and no clonal rearrangements of TCRβ, of α/δ gene locus, or the immunoglobulin gene in B lymphocytes. Findings of cerebrospinal fluid analysis were as follows: elevated protein, 0.92 g/L (0.15–0.45 g/L); negative Pan’s reaction; total number of red blood cells, 100 × 10^6^/L; total number of white blood cells, 4 × 10^6^ (0–8 × 106)/L; percentage of multinuclear cells, 0%; percentage of mononuclear cells, 1%; chlorine, 120.1 mmol/L (119–129 mmol/L); and glucose, 4.24 mmol/L (2.3–4.1 mmol/L). Red blood cells in the cerebrospinal fluid were considered as collateral injury, and cerebrospinal fluid cytological examination showed occasional lymphocytes, no abnormal cells, and few red blood cells. Flow cytometry studies of the cerebrospinal fluid were not performed. Moreover, the glucose level was 6.91 mmol/L, with glycated hemoglobin of 6.2% (4%–6%).

The patient was diagnosed as having SPTCL with CNS and cranial nerve involvement. The presence of lymph node involvement was not certain because the positron emission tomography-computed tomography examination was not completed; however, the physical examination and chest/abdominal and pelvic computed tomography found no lymph node involvement. After the first course of CHOP(cyclophosphamide, doxorubicin, vincristine, and prednisone)-like courses cyclophosphamide 800 mg on day 1, epirubicin 40 mg on day 1 and then 20 mg on day 2, vindesine 4 mg on day 1, dexamethasone 10 mg for days 1–5 chemotherapy, bilateral facial numbness, palsy, and drooping right eyelids considerably improved. On February 18, 2021, before the third course of CHOP-like course, she suddenly had progressive dyspnea and disturbance of consciousness. She was confirmed to have pulmonary consolidation and pleural effusion on chest computed tomography, and antifungal and anti-bacteria treatments were ineffective. The patient did not receive endotracheal intubation, and she died owing to respiratory and circulatory failure after 10 days. The disease progressed from clinical diagnosis to death in 66 days. A pathological biopsy of the lungs and autopsy was prohibited to respect the family members’ wishes.

## 3. Discussion

SPTCL was first described by Gonzales in 1991 as a disease with diverse clinical courses ranging from indolent to highly aggressive.^[3]^ Although it lacks significant atypia,^[6]^ its varying presentations^[5]^ makes its diagnosis a clinical challenge. The extracutaneous non-lymph node disease of SPTCL involvement of CNS, mesentery, or intraabdominal visceral fat, was usually negative on computed tomography, ultrasound, and magnetic resonance imaging.^[7]^ Positron emission tomography-computed tomography can be used to assess baseline cutaneous disease extent and treatment response.^[1]^ Apoptosis and autoimmune mechanisms, as well as genetic alterations, are involved in SPTCL.^[4,11,12]^ In addition, the overall death rate associated with SPTCL varied between 13% and 22%.^[3]^ CNS involvement in non-Hodgkin’s lymphoma is a fatal complication with a survival probability of <5%.^[13]^ Currently, there is no consensus regarding the most effective treatment for SPTCL. We summarized the basic futures of cases in the literature in Table 1.

**Table 1:** Xxxx

Summary of the basic features	
Clinical Features	
Frequency of primary cutaneous lymphomas	1%
5-year disease-specific survival	87%
Age	Median age 36, 20% of patients under 20 years
Sex	Female predominance
Anatomic site	Lower extremities and less commonly upper extremities and trunk
Skin lesion	Subcutaneous, indurated plaques and nodules; rarely ulcer, lipoatrophy and calcification
Systemic symptoms	“B” symptoms, Hemophagocytic syndrome
Preferred assess method	PET/CT
Therapeutic regimen	Steroids
	CHOP/CHOP-like
	Stem cell transplantation
	Immunosuppressive drugs
**Histologic and Immunophenotypic Features**	
Histologic features	Presents as lobular panniculitis of atypical small or medium lymphocytes with irregular hyperchromatic nuclei. Neoplastic lymphocytes form a rim around adipocytes, and fat necrosis, karyorrhexis, and cytophagocytosis
Immunophenotypic features	α/β T-cell phenotype, CD3+, CD4−, CD8+, TCRβF1+, Ki67+, Cytotoxic Proteins(+), CD30−, CD56+/−, Epstein–Barr virus-encoded small RNA-, TCR gene rearrangement seen in most cases

SPTCL is characterized by the expression of CD3 and CD8 since it is negative for CD4, CD30, CD56, TCRγ/δ, and Epstein-Barr virus, and positive for TCR-βF1.^[1,14]^ Patients with SPTCL are positive for CD2, CD5, and TiA1, with a high Ki-67 index.^[7]^ TCR gene rearrangement was performed as an adjunctive method to assist in the diagnosis of SPTCL for different clonal detections, varying from 50% to 90%.^[15]^ In our case, the specimen testing was performed after approximately 8 months led to a false-negative TCR gene rearrangement. The skin biopsy was positive for CD4, CD20, and CD30. CD20 is a specific B-cell marker, and primary CNS lymphoma is usually of B-cell origin. The incidence of the T-cell phenotype is only 1%–4%.^[16]^ This may suggest that our patient’s involvement with CNS is associated with diffuse positivity for CD20 and that the lymphomatous cells acquire preferential homing to cranial nerves. CD30 is a transmembrane glycoprotein receptor that is a member of the tumor necrosis actor receptor family,^[17]^ and it is noted in both reactive and neoplastic infiltrates.^[18]^ The phenotype of CD4+/CD8+ is a rare subtype of SPTCL as reported previously.^[15]^ Five in 9 cases show the CD4+/CD8+ phenotype, and no significant difference in clinicopathologic features was found when compared to the phenotype of CD4−/CD8+.^[7]^In another study, the proliferation marker Ki-67 staining ≥60% was observed in 72%–80% of patients,^[5,7]^which was similar to our case. This confirms that Ki-67 staining is a highly specific factor for diagnosing SPTCL.^[7]^Differential immunohistochemical characteristics were observed in SPTCL cases. This also prompted distinctive molecular profiles. However, whether the difference in immunohistochemistry suggests different clinical symptoms and prognosis needs to be further confirmed. In addition, molecular biomarkers may act as therapeutic targets for SPTCL.^[19]^ Rituximab is an anti-CD20 monoclonal antibody and an effective therapeutic antibody against systemic lymphoma,^[20]^ but Sluzevich et al described the case of a 76-year-old man who developed SPTCL after rituximab treatment for chronic lymphocytic leukemia in Mayo Clinic. The immunosuppressive effect of peripheral B-cell loss can be preferentially facultative to the development of selective T-cell lymphomas.^[21]^ In addition, the therapeutic benefit of rituximab in CD20+ T-cell lymphoma was unclear.^[22]^ Given the above considerations, we chose not to use Rituximab. Studies suggest that CD20 chimeric antigen receptor-modified T cells are effective against CNS lymphoma,^[23]^whose value in SPTCL with CD20+ and CNS involvement needs to be further investigated and approved.

The expression of CD20 in T-cell lymphoma is very uncommon, and the prevalence of CD20 expression in T-cell lymphoma was estimated to be between 5% and 8%.^[24]^ It was found that T-cell lymphoma with aberrant CD20 expression behaved in a very aggressive manner. In one study with 8 males who had a clinical follow-up, 5 died and 1 was recurrent, the remaining 2 were alive and undergoing treatment.^[22]^ A rare CD20+ primary central nervous system T-cell lymphoma patient treated with steroids, chemotherapy (vincristine and methotrexate), and rituximab, died 1 month after the initial diagnosis.^[24]^ The mechanisms of aberrant expression of B-cell lineage markers in T-cell lymphoma are unknown. CD20+ T-cell lymphoma may represent neoplastic transformation of an activated T-cell subset that has variable CD20 expression,^[22,25]^ whereas in other cases, CD20 may be an activation marker acquired after neoplastic transformation. CD20+ T-cell lymphoma can mimic marginal zone B-cell lymphoma in both nodal and extranodal sites, the enteropathy-type intestinal T-cell lymphomas microscopically simulated low-grade B-cell lymphoma of mucosa-associated lymphoid tissue type, particularly in cases that clinically and pathologically mimic B-cell lymphoma.^[22]^ In our case, the CD20+ SPTCL showed a significant ability to infiltrate into the CNS.

Chua et al reported about a patient with primary cutaneous T-cell lymphoma who presented with profound hearing loss and left facial nerve palsy after chemotherapy.^[26]^ Cranial magnetic resonance imaging did not detect any intraparenchymal lesions, and cerebrospinal fluid examination revealed lymphomatous leptomeningeal involvement. Jing Sun et al reported a case of SPTCL after chemotherapy that developed CNS involvement;^[10]^ a craniotomy biopsy showed CNS T-cell lymphoma, and TCR gene rearrangement showed TCR-β and TCR-γ clonal rearrangement. One SPTCL case developed Epstein-Barr virus-associated CNS B-cell lymphoma after alemtuzumab-CHOP treatment.^[27]^ These indicate that such patients may already possess subclinical CNS disease at the time as SPTCL diagnosis, and a small number of lymphoma cells may have existed in the CNS at presentation,^[13]^ indicating direct invasion of the tumor cells. Our patient had no history of chemotherapy, metabolic disorders, and vascular impairment before the onset of neurological symptoms. Direct invasion of lymphoma cells or immunological mechanisms (including paraneoplastic neuropathy) should be considered.

Paraneoplastic central nervous dysfunction is rare. Improvement of neuropathy after treatment of the tumor is a major criterion of the diagnosis of paraneoplastic disorders.^[28]^ Most paraneoplastic neuropathies occur at the onset of cancer or are the reason for its detection. The mechanisms of paraneoplastic diseases are heterogenous: autoimmune, metabolic, endocrine, and unknown. Autoimmune paraneoplastic syndromes are caused by onconeuronal antibodies and surface antibodies, but rarely in combination.^[29,30]^ Sensory neuronopathy is the most frequent type of paraneoplastic neuropathy.^[30]^ Cranial nerves V or VII can be a site of antero- and retrograde tumor spread,^[31]^ and neoplastic infiltration can spread from one cranial nerve territory to the other owing to anastomoses. The pyriform fossa is a site of transgression from one nerve territory into the other.^[32]^Local nerve infiltration can be erroneously diagnosed as Bell’s palsy or trigeminal neuralgia.^[33]^ Unfortunately, our patient never underwent gadolinium-enhanced head magnetic resonance imaging, indicating insufficient evidence of CNS infiltration. No abnormal autoimmune-related antibodies were found in the examination. Therefore, other related antibodies are unknown to properly diagnose the patient.

In summary, progressive spreading of symptoms with polycranial neuropathy and lung consolidation is highly suggestive of poor prognosis. The patient suddenly developed pulmonary consolidation; however, the source of the consolidation could not be investigated because lung biopsy was prohibited. The occurrence of infection was more related to the reduction of body immunity by multidrug chemotherapy. Despite treating the patient with antifungals and other antibiotics after having pulmonary lesions, the therapy still failed. This made the researchers assume that it was secondary to a lung lymphoma rather than an infection. Same as observed in our patient, SPTCL occurs with plaques, pulmonary consolidation, and fever, another case was diagnosed with SPTCL with lung involvement because her transbronchial lung biopsy showed perialveolar infiltration of predominant CD8+ T-cells.^[8]^ Upon diagnosis of this disease, early attention should be given to the lungs to avoid sudden respiratory failure and death. However, due to the short follow-up periods for many of the documented patients, the mortality rate is much higher than expected.^[2]^

Systemic dissemination to lymph nodes or other organs is rare and usually occurs late in the disease.^[34]^ The occurrence of hemophagocytic syndrome or nervous system and visceral involvement indicates progressive disease. The prognosis differs based on the stage of the disease. In the late stage of disease aggravation, the mortality rate is high. Therefore, careful medical history, systematic physical examination, early cerebrospinal fluid examination, early pathological biopsy, histopathology, and immunohistochemical staining are crucial for the accurate diagnosis and prompt treatment to reduce misdiagnosis and mortality due to SPTCL. Improving disease awareness is important.

## 4. Limitation

In the early stages, the skin lesion was neglected. Our patient never underwent gadolinium-enhanced head magnetic resonance imaging or positron emission tomography-computed tomography can.

## Author contributions

All authors contributed to the study conception and design. Jiajun Chen was the senior author of the report. Material preparation and the first draft of the manuscript were made by Jing Xu and all authors commented on previous versions of the manuscript. Data collection and analysis were performed by Jia Li, Ya-juan Sun, Wei Quan, Li Liu, Qing-hui Zhang, Yi-dan Qin, Xiao-chen Pei, and Hang Su. All authors read and approved the final manuscript.
